# The role of cats in the eco-epidemiology of spotted fever group diseases

**DOI:** 10.1186/1756-3305-7-353

**Published:** 2014-08-01

**Authors:** Ferran Segura, Immaculada Pons, Jaime Miret, Júlia Pla, Anna Ortuño, María-Mercedes Nogueras

**Affiliations:** Department of Infectious Diseases, Corporació Sanitària Parc Taulí - Institut Universitari Parc Taulí – Universitat Autonoma de Barcelona, Sabadell, Spain; Department of Medicine, Universitat Autonoma de Barcelona, Bellaterra, Spain; Animal Shelter Company (Vallès Oriental), Granollers, Spain; Veterinary Clinic “Centre”, Sabadell, Spain; Department of Animal Health, Universitat Autonoma de Barcelona, Bellaterra, Spain

**Keywords:** Spotted fever group Rickettsiae, *Rickettsia conorii*, *Rickettsia massiliae-*Bar29, *Rickettsia felis*, Cats, Molecular detection, Epidemiology

## Abstract

**Background:**

Mediterranean Spotted Fever (MSF), whose etiological agent is *R. conorii,* is one of the oldest described vector-borne infectious diseases. Although it is endemic in the Mediterranean area, clinical cases have also been reported in other regions. *R. massiliae-*Bar29 is related to MSF cases. This strain is distributed worldwide. *R. conorii* and *R. massiliae-*Bar29 are transmitted by ticks. Dogs are considered the sentinel of *R. conorii* infection. Cats could also be involved in their transmission. *Rickettsia felis,* etiological agent of Flea-borne spotted fever*,* is mainly transmitted by the cat flea, *Ctenocephalides felis*. Up to now, the role of cats in its transmission is not entirely elucidated. The aim of the study is to analyze the infection in cats by these microorganisms.

**Methods:**

The study was undertaken in Northeastern Spain. Twenty municipalities of seven regions participated in the study. 212 cats (pets and stray cats) were analyzed. Variables surveyed were: date of collection, age, sex, municipality, source, living place, outdoor activities, health status, type of disease, contact with other animals, and ectoparasite infestation. Sera were evaluated by indirect immunofluorescence antibody assay (IFA). Molecular detection (real-time PCR and sequencing) and cultures were performed on blood samples.

**Results:**

There were 59 (27.8%) cats seroreactive to one or more microorganisms. Considering cross-reactions, the seroprevalences were 15.6%-19.5% (*R. massiliae*-Bar29), 1.9%-6.2% (*R. conorii)*, and 5.2%-7.5% (*R. felis*). A weak association was observed between SFG seropositivity and tick infestation. Ticks found on seropositive cats were *Rhipicephalus pusillus, R. sanguineus* and *R. turanicus.* DNA of *Rickettsia* was detected in 23 cats. 21 of them could be sequenced. Sequences obtained were identical to those sequences of SFG rickettsiae similar to *R. conorii* and *R. massiliae*. No amplification of *R. felis* was obtained.

**Conclusions:**

Cats can be infected by SFG rickettsiae and produce antibodies against them. Cats may play a role in the transmission cycle of *R. conorii* and *R. massiliae*-Bar29, although the role in the *R. felis* cycle needs further analysis.

## Background

Spotted fever group (SFG) rickettsiae are Gram negative bacteria, obligate intracellular microorganisms, and widely distributed throughout the world. They are associated with arthropods, mainly with ticks, but also with fleas and mites. Some SFG rickettsiae can cause human disease. Clinical cases have been described in nearly every continent.

Mediterranean Spotted Fever (MSF) is one of the oldest described vector-borne infectious diseases. Many clinical cases have been reported since its etiological agent, *Rickettsia conorii,* was isolated in 1932. The usual vector is *Rhipicephalus sanguineus,* the brown dog tick [1]. This disease is endemic in the Mediterranean area [2] where *R. conorii* has also been isolated in different studies [3]. However, clinical cases have also been reported in other regions, such as Northern and central Europe, Northern Africa, Middle East, the Indian subcontinent and Asia [1, 4]. Moreover, *R. conorii* has also been detected in ticks from outside Europe. The main symptoms of MSF are fever and rash. A *tache noire*, arthralgia, myalgia, and headache are usually present [5]. Even though MSF is a benign disease in most cases, severe manifestations and cases of death have also been reported.

Initially, MSF had been considered the only tick-borne rickettsial disease in the Mediterranean area. However, new species have been described as a consequence of the improvement of molecular techniques. Some clinical cases of MSF showed different severity in manifestations and a different pattern of antibiotic sensitivity than those described classically [6]. As a consequence, it had been strongly suspected that other SFG rickettsiae could be involved in some MSF cases [1, 7]. In 1992, *Rickettsia massiliae* was isolated from ticks [1]. In 1996, the strain *R. massiliae-*Bar29 was isolated from ticks collected in our area [8]. A study conducted using sera from MSF patients suggested that *R. massiliae-*Bar29 could have been involved in cases of MSF [7]. In addition, sero-epidemiological studies showed *R. massiliae–*Bar29 past infection in humans [9, 10]. *R. massiliae* was confirmed as a human pathogen in 2006, when an isolate from an Italian patient, obtained 20 years before, was identified [11] *.* Up to now, two more human cases of *R. massiliae* infection have been described [12, 13]. *R. massiliae* strains are one of the most widely distributed *Rickettsia.* It has been described in all five continents. Nowadays, it is considered prevalent in America [1, 6, 8–25].

In 1990, *Rickettsia felis* was isolated from the flea *Ctenocephalides felis,* its main vector*. R. felis* is the etiological agent of Flea-borne spotted fever. Its main clinical manifestations are fever, headache, myalgia, and macular rash. In addition, severe manifestations have been described. Human clinical cases have been reported worldwide [1].

Prevention of zoonoses depends on detection of reservoirs, vectors, routes of transmission, and risk factors for infection. Although cats can be reservoirs of some microorganisms, they are very popular as pets around the world. Flea exposure is very frequent in cats [26]. Experimental cats exposed to *R. felis* infected fleas became seropositive and *R. felis* was detected in blood by PCR [27]. Naturally exposed cats were also seroreactive [27]. However, the role of cats in the *R. felis* transmission cycle has not been elucidated.

Nowadays, the dog is considered the sentinel of *R. conorii* infection [2, 28]. However, since cats can be exposed to ticks [26], antibodies against *R. conorii* have also been detected in cats [29–31]. Likewise, since *R. massiliae* strains have been found in *Rhipicephalus* spp. [6, 8, 12, 14–21, 24, 25], cats may also be seroreactive to this microorganism. To our knowledge, this fact has not been studied up to now.

In this study, we attempted to examine the antibodies against these three SFG rickettsiae in cats living in an area where these microorganisms are present [2, 3, 5, 8, 28, 32, 33]. In addition, we will analyze the possible infection of cats using molecular detection and culture.

## Methods

### Ethics statement

This study was approved by the Ethical Committee of Corporació Sanitària i Universitària Parc Taulí. This study was adherent to the Animal Protection Law (5/1995) of the Government of Catalonia, and RD1201/2005 of the Government of Spain, based on European Union directives 86/609/CEE and 2003/65/CE.

### Sample collection

The study was undertaken in Northeastern Spain. Twenty municipalities of seven regions participated in the study. Blood samples of 212 cats were collected from January 2001 to March 2009. Thirty-nine cats were stray cats that were kept as a strategy of control of rodent populations by municipalities. Their samples were provided by the municipal veterinarians when they carried out routine health care and reproductive controls. One hundred and seventy-one samples were collected at different veterinary clinics. One hundred and forty-seven of these cats were pets. One veterinary clinic worked together with Progat. Progat is a non-profit organization dedicated to the protection of stray cats, the sterilization of urban cat colonies, and the promotion of sterilization. This organization takes care of the health of stray cats. These cats undergo a veterinary examination when they are sterilized. In this study, 24 cats surveyed in veterinary clinics had been collected by Progat and, therefore, they were stray cats.

The following variables were registered: date of sample collection, age, sex, municipality, source (veterinary clinics, control of stray cats), living place (apartment, house, street), outdoor activities, health status, type of disease, contact with other animals, and ectoparasite infestation.

Blood samples were aseptically collected from the external jugular vein of each cat. One millilitre of blood was introduced in a serum-separating tube for serologic analyses, and the remainder was placed into a paediatric isolator 1.5 tube (Isolator™ 1.5; Oxoid, Ogdensburg, NY). Sera were obtained by centrifugation of blood at 1,500 rpm for 10 minutes. When it was possible, whole-blood samples were collected in sterile EDTA and heparin vacutainers. Samples were frozen at -80°C until used.

### Serological technique

Antibody titres against *Rickettsia conorii, Rickettsia felis*, and *Rickettsia massiliae-* Bar29 were evaluated by IFA using an anti-cat IgG (Sigma-Aldrich Química,S.A., Madrid). A commercial antigen (*R. conorii* spot, BioMérieux, Marcy l’Étoile, France) was used to determine antibodies to *R. conorii. R. felis* antigen was kindly provided by the Unité de Rickettsies, Marseille, France. *R.massiliae*-Bar29 antigen was obtained from that strain previously isolated from *Rhipicephalus sanguineus* in our region [8].

Briefly, 25 μL of twofold dilutions of cat sera in phosphate-buffered saline (PBS) – 3% non-fat dry milk were applied to the antigens. The slides were incubated in a humidified chamber at 37°C for 30 min. Two washes in PBS and one wash in water were performed to remove unbound immunoglobulins. Binding sera were detected using a fluorescein isothiocyanate-labelled anti-cat IgG (Sigma-Aldrich Química, S.A., Madrid) diluted 1/128 in PBS – 0.01% Evans Blue (Biomerieux, S.A., Madrid). The slides were incubated and washed as described above. The slides were examined with a fluorescence microscope at 400x. The highest dilution, at which distinct and specific fluorescence was seen, was scored as the end-point titre for the serum sample. The intensity of each specific fluorescence was evaluated and independently graded by two of the authors.

### Molecular detection

DNA from those samples collected in an EDTA vacutainer was obtained using the Masterpure DNA purification kit (Epicentre, Madison, Wisconsin) according to the manufacturer’s instructions. This kit was also used to obtain DNA from cultures of *R. conorii* and *R. massiliae-*Bar29 (obtained previously by our group, [3, 8]), and *R. felis*, kindly provided by the Unité de Rickettsies. These samples were used as positive controls. The DNA samples were stored at -20°C until used. Measures to avoid contamination were carried out using separate and dedicated rooms for DNA extraction and molecular detection.

The presence of a rickettsial agent was assessed by following real-time PCR assays: *R. felis*-specific PCR targeting the rickettsial gene for outer membrane protein B (*ompB*) [34], *Rickettsia*-specific PCR assays targeting 17 kDa antigen gene [35], *rickA* gene [36], and *ompA* gene. The *omp*A PCR assay, which was designed by our laboratory previously, amplified a fragment of 316 nucleotides within *omp*A gene (314-FOR: 5’-GGGCATTTACTTACGGTGGTGAT-3’; 630-REV: 5’-CTTTGACGGAGCTGCAGATTGTAT). The PCR targeting *rick*A and *omp*A genes used SYBGreen as an intercalant. The *Rickettsia*-specific PCR targeting 17 kDa gene and the *R. felis*-specific PCR targeting *omp*B gene used a probe. Sensimix dU kit (Quantance) was used. This kit included a Uracil DNA Glycosylase (UNG) and deoxyuracil triphosphates (dUTP) as well as a Hot start DNA polymerase. Concentrations used were as follows, 5 mM of MgCl_2_, 0.4 μM of probe and 0.5 μM of each primer for *omp*B PCR assay; and 5 mM of MgCl_2_, 0.4 μM of probe and 0.3 μM of each primer for 17 kDa PCR assay. Concentrations used in *omp*A PCR assay were 4.5 mM of MgCl_2_, and 0.2 μM of each primer. Concentrations used in *rick*A PCR assay were 5 mM of MgCl_2_, and 0.2 μM of each primer. Real-time PCR assays were carried out and analyzed using 7500 thermocycler (Applied Biosystems). PCRs were set up in a UV-sterilized workstation. Negative control consisted of PCR reagents and DNA-free water as template. Two negative controls and one positive control were included in all assays. Each sample was assayed twice.

Amplification products were purified by Exosap-it (GE Healthcare, Buckinghamshire, UK). DNA obtained was directly sequenced using forward and reverse primers. A new reverse primer was designed into the sequence amplified by the PCR targeting 17 kDa gene. For this purpose, FASTA sequences of 17 kDa genes of *Rickettsia* strains shown in Table 1 were obtained and aligned by CLUSTAL.W and Nucleotide Basic Local Alignment Search Tool - BLAST programs. Primers were designed using Primer BLAST program (NCBI). The primer selected was that located in a region where all sequences are identical (primer N8: 5’-TCCAACAAGCTGTCCTTTGCCCTT-3’). DNA sequencing was performed twice with each forward and reverse primers. When there was a sufficient amount of PCR product, this was also sequenced with primer N8. DNA was sequenced on a 3130 Genetic Analyser (Applied Biosystems) using a BigDye Terminator v3.1 Cycle Sequencing Kit (Applied Biosystems, Foster City, CA). Sequences obtained were compared with those in the GenBank nucleotide database by Nucleotide BLAST program. Sequences of cats were aligned with each other and with the rest of the *Rickettsia* strains (Table 1) using CLUSTAL.W and BLAST programs.

**Table 1:** ***Rickettsia*** **sequences used in this study**

Rickettsia	Sequence in GenBank (accession number)	Group ^a^
*R. massiliae*	*R. massiliae* MTU5 (CP000683.1), strain Alowo_68 (JN871729.1), strain AZT80 (CP003319.1), *Rickettsia* sp. TwKM01 (AY445821.1), Uncultured *Rickettsia* sp. clone 57 (GU353185.1)	Group-M
*R. africae*	*R. africae* ESF-5 (CP001612.1)	Group-C
*R. amblyommii*	*R. amblyommii* (AY375162.1), isolate AL-1 (EU828788.1), isolate TX051(EF689730.1), Candidatus *R. amblyommii* str. GAT-30 V (CP003334.1)	
*R. conorii*	strain Malish 7 (AE006914.1), RIRANT17KA *R.conorii* (M28480.1)	
*R. honei*	*R. honei* str. thai tick typhus (AF060706.1), *R. honei* (AF027124.1), strain RB (AF060704.1)	
*R. parkeri*	strain Portsmouth (CP003341.1), *R. parkeri* (U17008.1), isolate TX116 (EF689732.1), strain At24 (EF102237.1)	
*R. peacockii*	strain Rustic (CP001227.1), *R. peacockii* (AF260571.1)	
*R. philipii*	*R. philipii* str. 364D (CP003308.1)	
*R. rickettsii*	from Mexico (DQ176856.1), strain Hauke (CP003318.1), strain Hlp#2 (CP003311.1), strain Hino (CP003309.1), strain Iowa (CP000766.2), strain 'Sheila Smith' (CP000848.1), *R. rickettsii* (AY281069.1), strain Arizona (CP003307.1), strain Colombia (CP003306.1), strain Brazil (CP003305.1), strain ai103.1 (GU723477.1), strain ai101.1 (GU723476.1)	
*R. sibirica*	*R. sibirica* (AF445384.1)	
*R. slovaca*	clone 50 (JN182788.1), strain D-CWPP (CP003375.1), strain D-CWPP (CP003375.1), 13-B (CP002428.1)	
Candidatus *R. andeanae*	isolate T163 (GU395295.1)	
*Candidatus R. gravesii*	Candidatus *R. gravesii* (DQ269436.1)	
*Rickettsia sp.*	ARANHA (AY360215.1), 'Argentina' clone htrA_RArg_Apsd (EU826507.1), , COOPERI (AY362705.1), GRA-1 (AB444097.1), Hf332 (AB114804.1), , HpunctITA10 (AJ781417.1), HymargITA12 (AJ781419.1), RhturITA11 (AJ781418.1), R300 (AY472039.1), RpA4 (EF392727.1), scc31 (DQ105801.1), Is-1 (DQ344620.1), Tselentii (GU353184.1), TwKM03 (AY445822.1), DmargITA9 (AJ781416.1 ), HJ126 (ABAA4810.1), Ibadan (JN871831), Elepo (JN871731.1 )	
*Rickettsia* endosymbiont of	*A. maculatum* isolate TX012 (EF689728.1), *Carios kelleyi* (AY763102.1)	
Uncultured *Rickettsia sp.* clone	UnfedWild17.32 (GQ302897.1), UnfedWild17.10 (GQ302898.1), UnfedWild17.2 (GQ302894.1), FedWild17.57 (GQ302890.1), UnfedWild17.18 (GQ302896.1)	
*R. australis*	strain Cutlack (CP003338.1), RIRTRAPRO *R.australis* (M74042.1)	
*R. felis*	*R. felis* (GU447234.1), scc50 (DQ102709.1), URRWXCal2 (CP000053.1), 17 (AF195118.1), California (AF210693.1)	
*R. heilongjiangensis*	*R. heilongjiangensis* 054 (CP002912.1)	
*R. japonica*	*R. japonica* YH (AP011533.1)	
*R. marmionii*	strain KB (AY737683.1)	
*R. montana*	*R. montana* (U11017.1)	
*R. montanensis*	strain. OSU 85–930 (CP003340.1)	
*R. prowazekii*	*R. prowazekii* Rp22 (CP001584.1), strain GvF12 (DQ926851.1)	
*R. rhipicephali*	*R. rhipicephali* (U11020.1), strain HJ5 (DQ865207.1), *R. rhipicephali* (CP003342.1)	
*R. typhi*	strain Wilmington (AE017197.1), RIRANT17KB *R.typhi* (M28481.1 strain TH1527 (CP003397.1)	
Candidatus *R. antechini*	Candidatus *R. antechini* (DQ372953.1)	
Candidatus *R. hoogstraalii*	Candidatus *R. hoogstraalii* (FJ767736.1), (EF629538.1)	
*Rickettsia sp.*	HymargITA13 (AJ781420.1), HOT2 (AF483199.1), cf1and5 (AY953286.1)	
*Uncultured Rickettsia sp.*	clone FedWild17.12 (GQ302888.1), Clone Shimane 042 (AB699875)	

^**a**^Considering the region amplified by 17 kDa PCR, those sequences identical have been grouped.

### Culture

Cultures were carried out using the blood samples collected in the heparin vacutainers. For each animal, 150 microlitres of whole-blood was added to six shell vials (SVs) seeded with Vero cells (African green monkey epithelial cells). The SVs were centrifuged at 700 × g for 1 h at 22°C. The inoculum was discarded, and 1 mL of minimal essential medium (MEM) (Lonza, Basel, Switzerland), supplemented with 10% of fetal bovine serum (Lonza, Basel, Switzerland) and 1 mM of glutamine (Lonza, Basel, Switzerland), was added to each one. Three SVs were incubated at 32°C, and three at 28°C. Every week, the medium was replaced with a new medium.

After 5 weeks, a cell monolayer from each SV was scraped with glass beads and transferred to a confluent monolayer of Vero cells in a 25 cm^2^ cultured flask. Each flask was incubated at the same temperature used for SV incubation. Every week, a slide with medium and monolayer scraped from each flask was prepared for Gimenez staining.

After three weeks, cell monolayers were scraped with glass beads and cultures were collected. Cultures corresponding to the same cat incubated at the same temperature were joined. For each one, three slides were prepared for Gimenez staining and two IFA assays. One of the IFA assays was performed using a serum sample with antibodies against *R. conorii* (1/1024) and *R. felis* (1/1024). The other IFA assay was performed using a serum sample with antibodies against *R. massiliae-*Bar29 (1/256). Moreover, 0.8 mL of each cell monolayer scraped was used to obtain DNA. The presence of rickettsial DNA was analyzed using the PCRs described above.

### Statistical analysis

To achieve an accuracy of 5.0% in the estimation of a confidence interval using a normal asymptotic finite population correction for the bilateral 95%, assuming that the expected proportion was the highest prevalence found in the literature (worst cases) and that the total size of the populations were 1000, sample size was calculated.

The following variables were built: adult/kitten, demography, street, activities, and season of collection. The age was classified into categories [adult (≥1 year) and kitten (<1 year)]. The demographic area was determined considering the number of inhabitants of the municipality where the sample was collected. Municipalities with < 5,000 inhabitants were included in the rural area group, municipalities with 5,000 to 50,000 inhabitants were considered suburban areas, and municipalities with > 50,000 inhabitants were regarded as urban areas. The variable “Street” included stray cats and pets with outdoor activities. The variable “Activities” classified cats into three categories: indoor (pets without outdoor activities), outdoor (cats living at the street), indoor-outdoor (pets with outdoor activities).

Data analysis was carried out using the software application SPSS Statistics 18.0. A univariate analysis was performed to determine risk factors. Univariate group comparisons were performed using Chi-square and Fisher exact tests. Quantitative variables were compared by Mann–Whitney U test. A *p* < 0.05 was considered statistically significant.

## Results

### Study population

Samples were collected in twenty municipalities of seven regions. They are both urban and rural regions, as well as coastal and mountainous regions. The clinical and epidemiological characteristics of the study population are shown in Table 2. Of the 212 cats, 147 (69.3%) cats were pets and 65 (30.7%) stray cats. Outdoor activities were reported in 46 pets. Age ranged from 5 months to 17 years. The mean age was 3.7 ± 4.1 years. Sixty (29.1%) cats were kittens and 146 (70.9%) were adults. In six cats, age was not surveyed.

**Table 2:** **Demographic information from cats tested for antibodies to SFG rickettsiae**

Variable	Total population	Seropositive population ^a^
Age		
Kitten	60 (29.1)	16 (27.1)
Adult	146 (70.9)	43 (72.9)
Sex		
Male	99 (47.4)	23 (39.7)
Female	110 (52.6)	35 (60.3)
Demographical area		
Urban	172 (81.1)	46 (78)
Suburban	29 (13.7)	7 (11.8)
Rural	11 (5.2)	6 (10.2)
Habitat		
Apartment	53 (25)	14 (23.7)
House	94 (44.3)	24 (40.7)
Stray (PROGAT Foundation)	24 (11.3)	8 (13.6)
Stray cats controlled by municipalities	41 (19.4)	13 (22)
Activities		
Indoor	101 (47.6)	26 (44.1)
Outdoor	65 (30.7)	21 (35.6)
Indoor & Outdoor	46 (21.7)	12 (20.3)
Ectoparasites	65 (30.6)	20 (33.9)
Fleas	54 (25.5)	14 (23.7)
Ticks	12 (5.6)^b^	6 (10.2)^c^
Health status		
Healthy	156 (82.5)	40 (75.5)
Sick	33 (17.5)^d^	13 (24.5)^e^
Contact with animals	166 (78.3)	46 (77.9)
Contact with cats	129 (60.8)	38 (64.4)
Contact with dogs	48 (22.6)	15 (25.4)
Contact with other animals	5 (2.3)	0 (0)
Season of collection		
Winter	46 (21.7)	13 (22)
Spring	105 (49.5)	30 (50.9)
Summer	31 (14.6)	10 (16.9)
Autumn	30 (14.2)	6 (10.2)

^a^Cats with antibodies against one or more SFG rickettsiae (*R. conorii, R. felis, R. massiliae*-Bar29).

^b^ *Rhipicephalus pusillus* on 1 cat, *Rhipicephalus sanguineus* on 6 cats, *Rhipicephalus sanguineus* and *R. turanicus* on 1 cat, *R. sanguineus*, *R. turanicus* and *R. pusillus* on 1 cat. In two cats, tick specie was not determined.

^c^(*p* = 0.051) *Rhipicephalus pusillus* on 1 cat, *Rhipicephalus sanguineus* on 2 cats, *Rhipicephalus sanguineus* and *R. turanicus* on 1 cat, two ticks not identified.

^d^Dehydration, anaemia, cough, apathy, diabetes mellitus, hepatic diseases, respiratory diseases, Feline immunodeficiency, Feline leukaemia, breast lump, nasal lump, bacteraemia, cystitis, diarrhoea, fever, gingivitis, mouth infection, urinary tract infection, and uterus infection.

^e^Uterus infection, bacteraemia, fever, cough, listlessness, anaemia, respiratory disease, gingivitis, anaemia, dehydration, Feline immunodeficiency, and Diabetes mellitus.

Most samples were collected between March and July (65%). Samples of cats attended at veterinary clinics were collected throughout the year. All samples of stray cats controlled by municipalities and 82.6% of samples of stray cats from Progat foundation were collected between March and July.

Sixty-five (30.6%) cats were infested. Most of them were collected in April (12.7%), May (42.9%), and June (15.9%), (*p* < 0.001). There was higher proportion of infested cats among stray cats. In fact, 65.1% of stray cats have ectoparasites, whereas 12.4% of indoor pets and 23.9% of pets with outdoor activities had ectoparasites (*p* < 0.001). There was higher percentage of infested cats among those living in rural areas (63.6%) and suburban areas (50%) than among those living in urban environment (27.3%) (*p* = 0.006). Of infested cats, 98.4% had contact with animals (*p* < 0.001), 95% had contact with cats (*p* < 0.001), and 45.9% had contact with dogs (*p* = 0.016).

Fleas were found on 54 (25.5%) cats. Although most fleas (73.2%) were collected between February and July, no statistical association was found between infestation by fleas and month of collection. Ticks were collected on 12 (5.6%) cats. Ticks were identified as *Rhipicephalus pusillus, R. sanguineus,* and *R. turanicus.* Eleven ticks were collected in May and one in June (*p* < 0.001). All cats infested by ticks were stray cats controlled by municipalities in urban areas.

The health status was surveyed in 189 cats. Thirty-three (17.5%) cats showed some type of disease. These were: dehydration, anaemia, cough, apathy, Diabetes mellitus, hepatic diseases, respiratory diseases, Feline immunodeficiency, Feline leukaemia, breast lump, nasal lump, bacteraemia, cystitis, diarrhoea, fever, gingivitis, mouth infection, urinary tract infection, and uterus infection. Fifty percent of sick cats were stray cats (*p* = 0.030). A higher proportion of sick cats were found among infested cats (31%, *p* = 0.01) and cats with ticks (72.7%, *p* < 0.001).

### Sero-epidemiological study

Considering titres ≥ 1/64 as positive, there were 59 (27.8%) cats with antibodies against one or more of the antigens analyzed. Table 2 shows demographic information of these seropositive cats. A weak association was observed between SFG seropositivity and tick infestation (*p* = 0.051). Seropositive cats had a tick infestation rate twice as much as the overall study population (10.2% *vs.* 5.6%). Ticks found on seropositive cats were *Rhipicephalus pusillus, R. sanguineus* and *R. turanicus.*

Seropositive cats had a higher percentage of sick cats than the study population (24.5% *vs.* 17.5%). However, it was not statistically significant (*p* = 0.085). The diseases observed in seropositive cats were: Uterus infection, bacteraemia, fever, cough, listlessness, anaemia, respiratory disease, gingivitis, dehydration, Feline immunodeficiency, and Diabetes mellitus.

Thirteen (6.1%) cats had antibodies against *Rickettsia conorii* (1/64: 12 [5.6%], 1/128: 1 [0.5%]). Forty-one (19.3%) cats were seroreactive to *Rickettsia massiliae-*Bar29 (1/64:23 cats [10.8%], 1/128: 13 [6.1%], 1/256: 3 [1.4%], ≥ 1/512: 2 [1%]). In 16 cats (7.5%) antibodies against *Rickettsia felis* were detected (1/64: 14 [6.6%], 1/128: 1 [0.5%], 1/256: 1 [0.5%]). There were 11 (5.2%) cats with cross-reactions. It means that 19% of seropositive cats had antibodies against two or three species. Thirty-three (15.6%) cats were seropositive against *R. massiliae-*Bar29 exclusively, 4 (1.9%) cats against *R. conorii,* and 11 (5.2%) cats against *R. felis*. Cross-reaction between *R. massiliae-*Bar29 and *R. conorii* were observed in five cats, between *R. massiliae-*Bar29 and *R. felis* in 2 cats, and 3 cats presented cross-reactions between *R. conorii* and *R. felis.* One cat was seroreactive against the three species. Considering as a minimum value the percentage of cats seroreactive exclusively against each antigen, and the maximum value the percentage of all cats seroreactive against each antigen, seroprevalence of *R. massiliae*-Bar29 would range from 15.6% to 19.5%, seroprevalence of *R. conorii* from 1.9% to 6.2%, and that of *R. felis* from 5.2% to 7.5%. The titres as well as the clinico-epidemiological features of each seroreactive cat are shown in Table 3.

**Table 3:** **Information of seropositive cats**

*Cat’s* *number*	*17 kDa* *PCR* ^*a*^	*Titres*			*Habitat*	*Outdoor* *activity*	*Month of* *collection*	*Age* ^*b*^	*Sex* ^*c*^	*Demography* ^*d*^	*Ectoparasites* ^*e*^	*Sick* ^*f*^	*Contact with* *animals*
		*RC*	*RF*	*B29*									
***100***	+ (GM)	-	-	≥512	Apartment	Yes	January	17 y.	F	U	No	Bact	Cats, dogs
***102***	-	128	-	≥512	Apartment	No	February	4 y.	F	U	No	No	-
***50***	+ (GM,GC)	-	-	256	Apartment	Yes	May	6 y.	F	U	No	No	Cats
***89***	ND ^g^	-	-	256	Street	Yes	October	<1 y.	F	U	No	No	Cats
***104***	+ (GM,GC)	-	-	256	House	No	March	12 y.	F	U	No	No	Cats
***51***	+ (GM,GC)	64	-	128	Apartment	No	May	<1 y.	F	U	No	No	No
***54***	+ (GM)	64	-	128	House	Yes	May	6 y.	F	U	No	No	Cats
***57***	-	-	-	128	House	Yes	May	<1 y.	F	U	No	No	Gat, dogs
***61***	+ (GM)	-	-	128	House	No	June	1.8 y.	F	U	No	No	No
***67***	-	-	-	128	Street	Yes	June	2 y.	F	R	Fleas	No	Cats, dogs
***68***	+ (GM,GC)	64	-	128	Street	Yes	July	8 y.	F	R	No	No	Cats, dogs
***73***	-	-	-	128	House	Yes	May	1.2 y.	F	R	No	No	Cats
***77***	-	-	-	128	Apartment	No	July	9 y.	F	U	No	No	No
***83***	ND	-	-	128	House	No	September	6 y.	F	U	No	No	-
***103***	+ (GM,GC)	-	-	128	House	Yes	March	<1 y.	F	U	No	No	No
***181***	ND	-	-	128	Street	Yes	June	≥1 y.	F	U	No	No	Cats
***219***	-	-	-	128	Unknown	-	September	5 m.	F	U	Fleas	F, C, L	Cats
***260***	-	-	256	128	House	No	July	7 m.	M	SU	No	No	Cats
***4***	ND^e^	-	-	64	Apartment	No	January	5 y.	M	U	No	No	No
***7***	+ (GM,GC)	-	-	64	House	Yes	January	2 y.	M	U	No	No	Cats
***8***	ND	-	-	64	House	No	February	3 y.	M	U	No	No	Cats
***24***	+ (GM)	-	-	64	Street	Yes	February	<1 y.	F	U	Fleas	No	Cats, dogs
***33***	-	-	-	64	Apartment	No	March	13 y.	F	U	No	No	Dogs
***47***	+ (GM,GC)	64	64	64	House	No	May	<1 y.	F	U	No	No	Cats
***53***	-	-	-	64	House	Yes	May	<1 y.	M	U	Fleas	No	Cats
***66***	+	64	-	64	Street	Yes	June	<1 y.	F	R	No	No	Cats, dogs
***69***	-	-	-	64	Street	Yes	July	2 y.	F	R	Fleas	Ov Inf	Cats, dogs
***76***	-	-	-	64	House	Yes	July	4 y.	M	U	No	No	Cats
***98***	+ (GM)	-	-	64	House	No	December	1.4 y.	F	U	No	No	Cats
***105***	-	-	-	64	House	No	March	2.6 y.	M	U	No	No	-
***111***	+ (GM,GC)	-	-	64	Street	Yes	April	1 y.	F	U	No	No	Cats
***183***	ND	-	-	64	Street	Yes	June	≥1 y.	M	U	Ticks	No	Cats
***207***	-	-	-	64	House	Yes	April	7 m.	F	SU	Fleas	No	Cats
***209***	GM	-	-	64	Apartment	No	April	11 m.	M	SU	No	No	Cats, dogs
***216***	-	-	-	64	Apartment	No	May	9 m.	M	U	Fleas	-	No
***217***	+ (GM,GC)	-	-	64	House	No	May	8 m.	M	R	Fleas	No	Cats
***233***	ND	-	-	64	Street	Yes	May	≥1 y.	F	U	RS	Res Dis	Cats
***243***	ND	-	64	64	Street	Yes	May	≥1 y.	M	U	RS	Res Dis	Cats
***247***	ND	-	-	64	Street	Yes	May	≥1 y.	M	U	RP	Res Dis	Cats
***266***	-	-	-	64	Apartment	Yes	June	9 y.	M	SU	Fleas	FIV	Cats, dogs
***275***	+ (GM)	-	-	64	Apartment	No	October	7 m.	F	U	No	No	No
***13***	+ (GM)	-	64	-	House	No	January	3 y.	M	SU	Fleas	No	No
***42***	+ (GC)	64	**128**	-	Apartment	No	April	7 y.	M	U	No	No	No
***52***	+ (GM,GC)	64	-	-	Apartment	No	May	3 y.	M	U	No	No	No
***58***	+	-	64	-	Apartment	No	May	<1 y.	M	U	No	No	No
***94***	-	64	64	-	House	No	November	<1 y.	F	U	No	No	Cats, dogs
***97***	-	64	-	-	House	Yes	December	3 y.	F	SU	Fleas	No	Cats, dogs
***170***	-	-	64	-	Street	Yes	March	≥1 y.	M	U	Fleas	Res Dis	Cats
***171***	+ (GM,GC)	64	64	-	Street	Yes	March	≥1 y.	F	U	No	No	Cats
***175***	ND	64	-	-	Street	Yes	May	≥1 y.	F	U	Ticks	NR	Cats
***179***	ND	-	64	-	Street	Yes	June	≥1 y.	F	U	No	No	Cats
***180***	ND	-	64	-	Street	Yes	June	≥1 y.	F	U	No	No	Cats
***193***	-	-	64	-	Apartment	No	January	9 y.	F	U	No	Res Dis	gos
***198***	+ (GM,GC)	-	64	-	House	No	February	13 y.	F	SU	No	An, Deh	Cats, dogs
***237***	ND	64	-	-	Street	Yes	May	≥1 y.	F	U	RS,RT	Ging	Cats
***240***	ND	-	64	-	Street	Yes	May	≥1 y.	F	U	Fleas	No	Cats
***252***	ND	-	64	-	Street	Yes	May	≥1 y.	F	U	Fleas	No	Cats
***274***	ND	-	64	-	House	No	October	7 y.	F	U	No	Diab	No
***272***	ND	-	64	-	House	Yes	July	1 y.	M	U	No	No	Cats

^a^Sequence homology are shown in brackets. GM: identical to those sequences joined as group-M in Table 1; GC: identical to those ones joined as Group-C. ^b^y: years; m: months. ^c^F: female, M: male. ^d^U: urban, SU: suburban; R: rural. ^e^RT: *Rhipicephalus turanicus*, RS: *Rhipicephalus sanguineus*, RP: *Rhipicephalus pusillus*. ^f^Bact: Bacteraemia; F: Fever; C: cough; L: listlessness: Ov Inf: Ovarian infection; Res Dis: Respiratory disease; FIV: Feline Immunodeficiency; NR: Cat ill but disease not reported An: Anaemia, Deh: dehydration; Ging: Gingivitis; Diab: Diabetes. ^g^ND: Not done.

### Molecular detection

Whole blood samples had been collected in EDTA-vacutainers in 42 of the 59 seropositive cats. No amplification was obtained when PCRs targeting genes *omp*A, *rick*A, and *omp*B were performed. When a 17 kDa PCR assay was carried out, positive amplifications were observed in 23 cats (Table 3). The amplification products were sequenced. The amount of DNA amplified was not enough to analyse it by sequencing in two cats (numbers 58, and 66). Low copy number of rickettsial DNA amplified as well as the short length of amplicon (141 nucleotides) allowing us to obtain good short sequences. Thus, the sequence consensus for each cat was defined as that fragment in which two or more sequences obtained align to each other.

As 17 kDa PCR assay was the only PCR with positive results, in order to be sure of the amplifications, DNA from 29 seronegative cats was extracted and this PCR was performed using those DNAs as templates. No amplification was obtained in these 29 samples.

**Figure 1:**
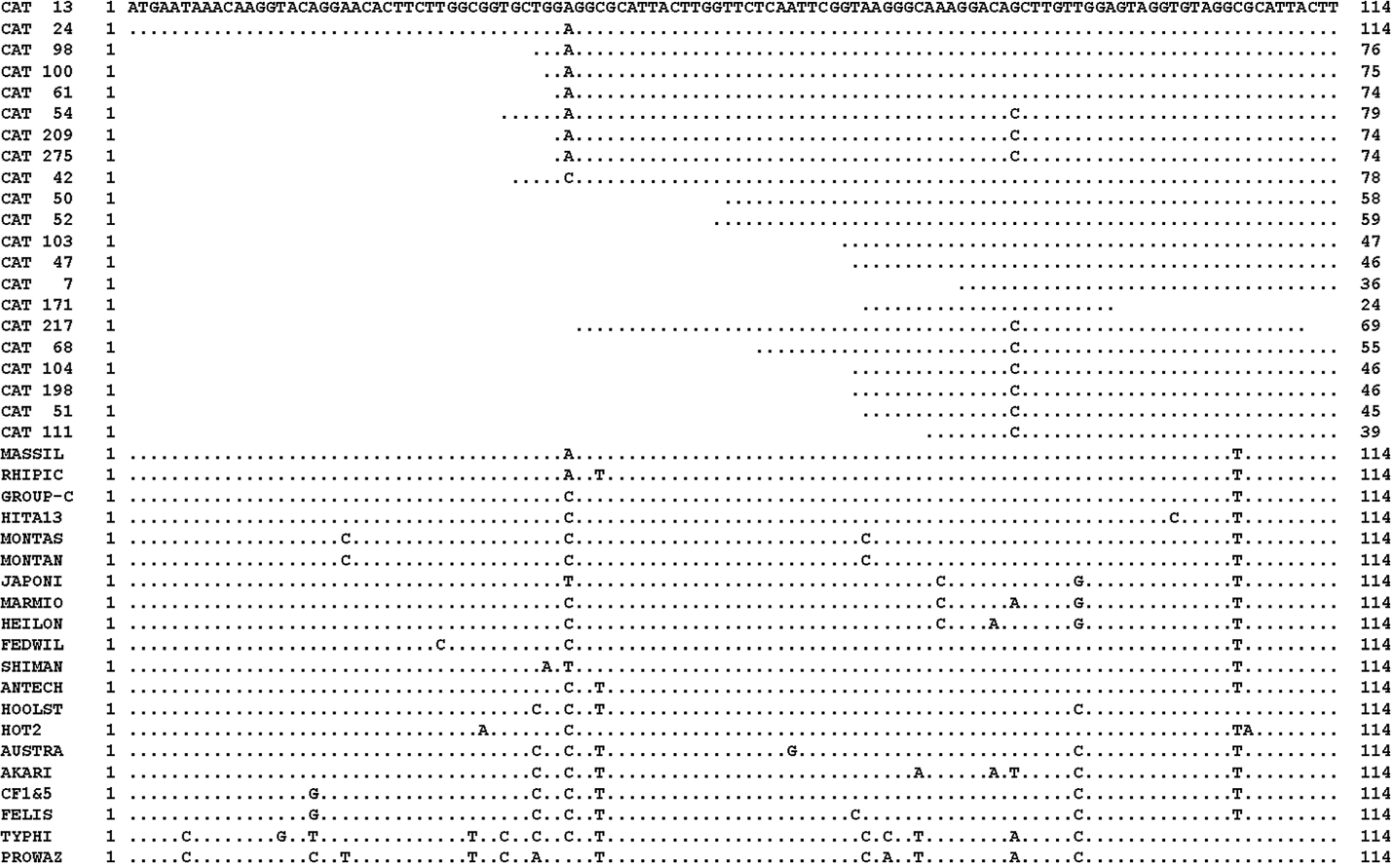
**Alignment of the fragments amplified by 17 kDa PCR. MASSIL:** Massiliae Group-M (see Table 1) **RHIPIC:** R. rhipicephali **Group-C** (group of sequences identical among them, see Table 1) **HITA13:** Rickettsia sp. HymargITA13, **MONTAS:** R. montanensis, **MONTAN:** R. montana, **JAPONI:** R. japonica, **MARMIO:** R. marmionii, **HEILON:** R. heilongjiangensis, **FEDWIL:** Uncultured Rickettsia sp. clone FedWild17.12, **SHIMAN:** Uncultured Rickettsia sp. clone Shimane042, **ANTECH:** Candidatus R. antechini, **HOOLST:** Candidatus R. hoogstraalii, **HOT2:** Rickettsia sp. HOT2, **AUSTRA:** R. australis, **AKARI:** R. akari, **CF1&5:** Rickettsia sp. cf1and5, **FELIS:** R. felis, **TYPHI:** R. typhi, **PROWAZ:** R. prowazekii.

The 17 kDa PCR amplifies a fragment of 114 nucleotides. All *Rickettsia* strains that belonged to the same species had identical sequences into this fragment. For this study, sequences were joined into groups (Group-M and Group-C) when they were identical among them (Table 1). Figure 1 shows alignment of the different *Rickettsia* sequences found in this region, as well as all consensus sequences obtained from the cats. All sequences obtained were similar to Group-M or Group-C rickettsiae and different to the other ones. One nucleotide allows differentiating Group-M and Group-C. Nine cats had this nucleotide included in their consensus sequences. One cat had a ‘C’ like Group-C*.* Eight cats had an ‘A’ as Group-M*.* In 11 cats, it was not possible to define the different nucleotide between Group-M and Group-C. Table 4 showed the results of comparing consensus sequences of each cat with those in the GenBank by Nucleotide BLAST program.

**Table 4:** **Results obtained using database of Basic Local Alignment Search Tool (BLAST)**

Cat	Sequence length	Highest homology ^a^	Score	Max. Identity	Identities
13	114	Group-M	201	99%	113/114 (99%)
24	114	Group-M	201	99%	113/114 (99%)
98	76	Group-M	132	99%	75/76 (99%)
100	75	Group-M	131	99%	74/75 (99%)
61	74	Group-M	129	99%	73/74 (99%)
54	79	Group-M	134	97%	77/79 (97%)
209	74	Group-M	125	97%	72/74 (97%)
275	74	Group-M	125	97%	72/74 (97%)
42	78	Group-C	139	99%	77/78 (99%)
50	58	Group-C or Group-M	102	98%	57/58 (98%)
52	59	Group-C or Group-M	102	98%	58/59 (98%)
103	47	Group-C or Group-M	85.7	98%	46/47 (98%)
47	46	Group-C or Group-M	83.8	100%	45/46 (98%)
7	36	Group-C or Group-M	63.9	100%	35/36 (97%)
171	24	Group-C or Group-M	48.1	100%	24/24 (100%)
217	69	Group-C or Group-M	116	97%	67/69 (97%)
68	55	Group-C or Group-M	91.5	96%	53/55 (96%)
104	46	Group-C or Group-M	75.8	96%	44/46 (96%)
198	46	Group-C or Group-M	75.8	96%	44/46 (96%)
51	45	Group-C or Group-M	73.8	100%	43/45 (96%)
111	39	Group-C or Group-M	61.9	100%	37/39 (95%)

^a^Group-M: those sequences in GenBank identical to *R. massiliae;* Group-C: sequences in GenBank that belong to different *Rickettsia* sequences (including *R. conorii*) and are identical among them (Table 1).

### Culture

Cats selected were those in which rickettsial DNA was detected in blood samples, or whose titres of antibodies against *R. conorii*, *R. felis*, or *R. massiliae-*Bar29 were ≥ 1/128. Among them, cultures were carried out in those cats whose heparin whole-blood samples were available (Cats: 181, 198, 260, and 275). All six SVs of cats 275 and 181 were incubated at 32°C. Three SVs of cats 198 and 260 were incubated at 28°C, whereas three other ones were incubated at 32°C. After 3 weeks of incubation in a 25 cm^2^ flask, all cultures were negative by molecular detection and IFA.

## Discussion

Cats can be the reservoir of many pathogens. A considerable percentage of cats are pets, which are in close contact with humans. Therefore, it is important to perform serological as well as molecular studies of pathogens in cats. It was suggested that cats can be sentinels for rickettsiae [30]. However, their role in maintaining and transmitting these microorganisms to humans has not been entirely elucidated [37]. Our data showed a substantial percentage (27.1%) of SFG seropositive cats, even with high titres and molecular detection in blood. Some studies have also shown that cats can be seroreactive to SFG rickettsiae [29–31, 38, 39]. For instance, more than 50% of cats studied in Australia were SFG seroreactive [38]. Cross-reaction among SFG rickettsiae has been well described and it is a limitation of seroprevalence studies. We used antigens of those SFG rickettsiae described in our area [2, 3, 5, 8, 28, 32, 33]. In our study, a proportion of cats were seropositive exclusively against one of the SFG rickettsiae studied. Therefore, although actual seroprevalences couldn’t be established, data do show seroreactivity of cats against each antigen. Taking into account cross-reactions, *R. massiliae*-Bar29 seroprevalence was 15.6% - 19.5%, *R. conorii* seroprevalence was 1.9% - 6.2%, and *R. felis* seroprevalence was 5.2% - 7.5%.

Interestingly, *R. massiliae*-Bar29 seroprevalence was the highest, even if cats with antibodies exclusively against this strain were considered (15.6%). Moreover, highest titres were observed against *R. massiliae*-Bar29 antigen, most of them in cats without cross-reactions. The majority of studies are focused on the presence of *R. massiliae* strains in ticks. In fact, *R. massiliae* has been detected in *Rhipicephalus sanguineus, R. turanicus, R. pusillus, R. guilhoni, R muhsamae, R. lunulatus, R. sulcatus* collected on humans, dogs, cats, donkeys, cows, hedgehogs, horses, red foxes, roe deer, goat, cattle and sheep and wild boars or by flagging [6, 8, 12, 14–21, 24, 25]. Moreover, it has been found in other genera such as *Ixodes tasmani* (collected on Tasmanian devils) [22], *Ixodes ricinus* [16], and *Dermacentor marginatus* (collected on dogs) [23]. Association between ticks and human cases has been observed [12, 16, 24]. The transstadial and trasovarial transmission of *Rickettsia massiliae-*Bar29 have been observed in *R. sanguineus* group. In addition*,* this microorganism does not have any effect on viability of *R. sanguineus* and its reproductive fitness [6, 25, 40]. Therefore, these ticks are considered a reservoir.

On the other hand, in spite of the fact that mammals can be reservoirs for many *Rickettsia* species, little is known about the role of them in the *R. massiliae* cycle. Since *Rhipicephalus* spp. is described mainly as a dog tick, most studies are focused on dogs. For instance, association between *R. massiliae-*Bar29 seropositivity in humans and contact with dogs had been observed in our previous serological study [9]. *R. massiliae* infection had been detected by serum cross-absorption and Western blot in dogs from California [25]. To our knowledge, this is the first study focused on *R. massiliae-*Bar29 infection in cats.

Tick infestation was observed in our cats. All of the ticks identified belonged to the genus *Rhipicephalus (R. sanguineus, R. pusillus*, and *R. turanicus),* whose members can use cats as possible hosts [26]. Cats showed lower infestation by ticks than by fleas. On the one hand, the lower infestation by ticks could be due to the host specificity because of *Rhipicephalus* spp. tends to infest mainly dogs. On the other hand, some authors explain the low infestation by ticks as a consequence of the grooming habits of cats [41]. In addition, since most cats in our study were pets, they were probably disinfested by their owner. In fact, all cats infested by ticks were stray cats surveyed by municipal veterinarians. Considering our area as an endemic area for the *R. massiliae-*Bar29 strain [7–9], it can be suspected that this microorganism may infect some of the ticks on the cats. In fact, *R. massiliae* had been detected in one *R. turanicus* collected on a cat in the Camargue [14]. Some studies have described cats not only as transport hosts of ticks, but also as susceptible and seroreactive to infection of SFG rickettsiae [30, 31]. According to our results, cats can be infected and produce antibodies against *R. massiliae-*Bar29. More than fifteen percent of our cats were seroreactive against *R. massiliae-*Bar29, exclusively. Moreover, high titres were observed. For instance, cat number 102 had titres higher than 512 (more than two-fold of its *R. conorii* titre) and cat number 100 had antibodies against exclusively *R. massiliae-*Bar29 at titres higher than 512.

The role of *R. sanguineus* in the transmission of *R. conorii* has been widely described since 1930 [1]. A transstadial and trasovarial transmission of *R. conorii* takes place in this tick. Therefore, *R. sanguineus* cannot only act as a vector but also as a reservoir [30]. However, unlike *R. massiliae, R. conorii* can negatively affect the survival and fecundity of *R. sanguineus* [6, 40] *.* For this reason, *R. conorii* in ticks declines gradually each generation [40]. This could explain why the prevalence of *R. conorii* is usually low in ticks. In fact, whereas *R. massiliae* has been detected, *R. conorii* has not been found in ticks from areas where both species have been described [8, 16, 17]. As a consequence, the role of the mammals as reservoir hosts is important. Epidemiological surveys have demonstrated the role of dogs, sheep and hedgehogs as vertebrate reservoirs for *R. conorii* [2]. High seroprevalence in dogs has been observed in MSF endemic areas [4]. Dogs are considered reservoirs and sentinels of *R. conorii* [28, 42] *.* Recently, *R. conorii* has been detected in blood samples of dogs by PCR and sequencing [43]. On the other hand, some studies have shown that cats can be seroreactive to *R. conorii* [29, 38]. A Spanish study showed that 44% of cats were seroreactive against *R. conorii* antigen. Titres ranged from 64 to 8192 [31]. In a study carried out in Zimbabwe and South Africa, 34% and 19% of cats had antibodies against *R. conorii* [30] *.* We did not use only *R. conorii* antigen but antigens of other SFG rickettsiae of our area. Almost 2% of our cats had antibodies against *R. conorii,* exclusively. Therefore, seroreactivity of cats to *R. conorii* is confirmed.

Seroprevalence studies suggest the exposure of the animal to microorganisms. However, the infection needs to be shown by the detection of the microorganisms within the animal. DNA of SFG rickettsiae were detected in blood of our cats. Three PCR assays that amplify SFG rickettsiae were used (17 kDa, *rick*A, and *omp*A). Positive results were only obtained using real time PCR targeting the 17 kDa gene. The latter incorporated a probe while *rick*A and *omp*A used SYBGreen as intercalant. Real time PCR using probes instead of SYBGreen are much more sensitive. The amount of *Rickettsia* in blood may have been very low as it was only detected by 17 kDa PCR. In fact, in two cases, sequencing was not possible due to the low amount of DNA amplified. In order to be sure of these results, DNA from 29 seronegative cats was used as a template for 17 kDa assays and no amplification was obtained.

The sequence amplified by 17 kDa assay is not able to distinguish among all SFG rickettsia. This is a limitation of the study. DNA detected belonged to two groups of SFG rickettsiae. In some cats, rickettsiae closely related to *R. massiliae* were identified pointing towards the presence of its infection in cats. It is important to highlight that most cats, in which rickettsia DNA were detected, were pets. Therefore, SFG rickettsiae may be in close contact with humans through the cats. However, the role of cats as reservoirs of these microorganisms needs further studies to focus on the cats’ ability to acquire the microorganisms from vectors, maintain or amplify them, and transmit them again to vectors.

*Ctenocephalides felis* is the main vector of *R. felis,* although this microorganism has been detected in other fleas [32]. In addition, fleas act as a reservoir for *R. felis* because vertical transmission has been observed [27]. Experimental infection of cats has been demonstrated when they have been exposed to fleas infected with *R. felis.* The experimentally infected cats became seroreactive by the fourth month after exposure, had a very short bacteraemia, and *R. felis* DNA was detected in blood transiently [27]. However, the role of the cat in the epidemiology of *R. felis* has not been determined yet. *R. felis* was detected in 43.6% of fleas (*Ctenocephalides felis*) from our area [32]. In our study, a quarter of cats had fleas. *R. felis* seroprevalence in cats ranged from 5.2% to 7.5%. Likewise, some studies found serum antibody responses to *R. felis* in naturally infected cats [37, 39]. Therefore, naturally infected cats are susceptible to *R. felis* infection and produce antibodies against it.

Interestingly, whereas DNA closely related to other species of SFG rickettsiae was detected in blood samples, no DNA related to *R. felis* was identified. Amplification was obtained by PCR targeting 17 kDa using a probe. Although this PCR amplifies a short fragment of DNA, its sequencing would allow the identification of *R. felis.* However, no sequence obtained was similar to those of *R. felis* in GenBank. In the same way, *R. felis*-specific PCRs targeting the *ompB* gene were negative. Quite a few studies have failed to detect this microorganism in blood [37, 44–46]. For instance, whereas *R. felis* was detected in 18% of fleas from Ontario, molecular detection was negative in cats [45]. When pairs of feline blood and fleas were analyzed by Hawley *et al*., 67.4% of fleas were infected with *R. felis*; however, no cat was positive [46]. Bayliss *et al*. studied cats with and without fever. Although both cat populations were seropositive, no *R. felis* was detected in any blood sample [37]. There are some hypotheses about the lack of *R. felis* amplification in blood. It could be related to a rapid immune response [37]; a low concentration of *R. felis* in blood, lower than the detection limit of PCR assays [44, 46]; an intermittent bacteraemia that could be missed when the sample is taken [47]; and a sequestration of *R. felis* in other tissues such as endothelial cells, spleen, and dermal tissues [44, 46]. In this way, Lappin *et al*. analyzed the presence of *Rickettsia* DNA in blood, oral cavity, skin, and claw beds of 83 dead cats using the same PCR of Hawley’s study. *R. felis* DNA was detected in skin and gingival samples [47]. In the same way, *R. felis* were detected in renal, hepatic and pulmonary tissues of opossums while PCR of blood samples were negative [48]. Therefore, although cats can be susceptible to *R. felis* infection, probably they may not be an effective reservoir. A high percentage of our cats were pets in close contact with humans. In our area, *R. felis* prevalence in fleas is much higher that *R. felis* seroprevalence in the human population [32, 33] *R. felis* may be maintained in a cycle away from humans who were sporadically infected. Moreover, cats may be an unlikely reservoir host and may not have an important role in its transmission.

Overall, there were no significant associations between seropositivity and either habitat or outdoor activities, even though stray cats or cats with outdoor activities might be more exposed to infection. Similar results have been described in rickettsiae as well as other pathogens infecting cats [39, 44]. Our results may be due to the fact that our area is a predominantly urban area, in which stray cats are very controlled, and most of them are sterilized. On the other hand, there were more pets than stray cats in our survey. As a consequence, seropositive cats, even cats in which rickettsiae have been directly detected, lived in close contact with humans.

Like other studies [31], sex and age were not associated to SFG seropositivity. No statistically significant association was found between SFG seroprevalence and health status. Therefore, cats may present a SFG rickettsiae subclinical infection. Likewise, experimentally *R. felis* infection in cats was asymptomatic [27]. Moreover, those cats, in which *R. felis* had been detected in oral cavity or skin by PCR, did not have clinical evidence of skin disease or gingivitis [47]. Bayliss *et al.* did not find statistically significant association between fever and both *R. felis* and *R. rickettsii* seropositivity [37]. In the same way, there was no association between health status and *R. conorii* seroprevalence in the Solano’s study [31]. Izzard *et al*. did not find statistical correlation between illness and SFG seropositivity [38]. These authors also suggested that clinical symptoms could be so early and mild that they may not warrant veterinary attention.

Even though the highest percentage of infested cats and stray cats were surveyed between March and July, there was no statistically significant association between month of the year and SFG seropositivity. On the one hand, it could be due to the persistence of antibodies over time. On the other hand, larvae and nymphs can also be vectors of SFG rickettsiae [1]. Eggs, larvae, nymphs and unfed adults have also been collected in the winter [40]. Taking into account ticks feed at each life stage as well as transstadial and transovarial transmission [1], rickettsiae can be transmitted to the host in each season. Moreover, early stages are smaller than adults and could go unnoticed. Finally, although *R. sanguineus* is active from spring to autumn, the climate change could be influencing its activity.

## Conclusions

In conclusion, cats can be infected by SFG rickettsiae and produce antibodies against them. In spite of cross-reaction, some cats have reacted exclusively against one of the species studied: *R. conorii, R. massiliae-*Bar29, and *R. felis.* Although a good identification of species was not possible by molecular detection, infection by SFG rickettsiae has been observed; even a strain closely related to *R. massiliae-*Bar29. Therefore, cats may have a role in the transmission cycle of these microorganisms, particularly in those such as *R. conorii,* which can be decline gradually in tick populations. *R. felis* has not been detected in cat blood samples, pointing towards *R. felis* being found in other tissues, in addition, cats may not have a main role in its transmission cycle. Further studies will be necessary to study in depth the importance of cats as reservoirs of SFG rickettsiae. This fact is particularly important because many of them live in close contact with humans, and could be infected in spite of their habitat, infestation, month of year, or health status.

## Abbreviations

BLAST: Basic Local Alignment Search Tool
DNA: Deoxyribonucleic acid
dUTP: deoxyuracil triphosphates
EDTA: Ethylenediaminetetraacetic acid
IFA: indirect immunofluorescence antibody assay
MSF: Mediterranean Spotted Fever
NCBI: National Center for Biotechnology Information
PCR: Polymerase chain reaction
SFG: Spotted fever group
SV: Shell vial (shell vials – SVs)
UNG: Uracil DNA Glycosylase.
